# Stoichiometric Characteristics of Carbon, Nitrogen, and Phosphorous and Allometric Nitrogen–Phosphorous Relationships During the Organ-to-Forest Floor Material Transformation in Representative Forest Tree Species on the Southern Slope of the Qilian Mountains

**DOI:** 10.3390/biology15131014

**Published:** 2026-06-26

**Authors:** Xukai Yang, Shuang Ji, Jiaxiang Xu, Xiaoping Kong, Yinglian Qi, Yue Zhang, Huichun Xie, Jiawei Yan

**Affiliations:** 1 School of Life Sciences, Qinghai Normal University, Xining 810008, China; 13516177294@163.com (X.Y.);; 2 College of Geographical Sciences, Qinghai Normal University, Xining 810008, China; 3 Qilian Mountain Southern Slope Forest Ecosystem Research Station, Haidong 810500, China; 4 Key Laboratory of Medicinal Animal and Plant Resources of Qinghai-Tibetan Plateau, Xining 810008, China; 5 Team of Germplasm Resources Formation Mechanism and Utilization on the Qinghai-Tibetan Plateau, Xining 810008, China; 6 Xining Botanical Garden, Xining 810008, China

**Keywords:** Qilian Mountains, ecological stoichiometry, forest floor material, allometric relationship, nutrient limitation

## Abstract

The ecological stoichiometric traits of forest floor material nutrient resorption in forest ecosystems. However, systematic insights into nutrient allocation and scaling during the transformation of plant organs to forest floor material remain limited. This study examined six representative tree species on the southern slope of the Qilian Mountains, quantifying the carbon (C), nitrogen (N), and phosphorus (P) contents and their stoichiometric characteristics in leaves, branches, and mixed forest floor material. Allometric relationships between N and P were analyzed using the standardized major axis regression. Coniferous species exhibited a conservative strategy with high C/N and C/P ratios, whereas broad-leaved species and mixed forests exhibited nutrient-enrichment strategies. During the organ-to-forest floor material transformation, N and P contents significantly reduced, whereas C/N and C/P ratios increased, indicating strong nutrient resorption. N and P were positively associated with plant organs and forest floor material, with isometric relationships (b = 1.06 and 0.98 for plant organs and forest floor material, respectively). Because the slopes did not differ significantly from 1 (*p* > 0.05), the N:P ratio remained relatively constant, with no significant P limitation. This indicates that tree species regulate forest floor material quality through divergent nutrient utilization strategies, modulating nutrient cycling. This study provides a theoretical foundation for the ecological restoration and stand structure optimization of alpine forests.

## Simple Summary

Forest ecosystems on the southern slopes of the Qilian Mountains play an important role in regional ecological stability and nutrient cycling. However, it remains unclear how carbon, nitrogen, and phosphorus are distributed among plant organs and forest floor material in different tree species. In this study, we compared the C, N, and P concentrations and their stoichiometric ratios in leaves, branches, and forest floor material of six representative tree species. We found clear differences among species and between plant organs and forest floor material. Broad-leaved species generally had higher nutrient concentrations, whereas coniferous species showed higher C/N and C/P ratios. During the transition from plant organs to forest floor material, nitrogen and phosphorus concentrations decreased, while C/N and C/P ratios increased. These results suggest that nutrient resorption before leaf fall and differences in litter quality may strongly influence nutrient cycling in these forests. Our findings provide useful information for understanding plant–litter nutrient relationships in alpine forest ecosystems.

## 1. Introduction

Ecological stoichiometry provides a crucial theoretical framework for understanding nutrient cycling and limitations in ecosystems by elucidating the relationships among carbon (C), nitrogen (N), and phosphorus (P) between organisms and their environment [1,2]. As core elements underlying plant growth and ecological processes, stoichiometric traits are crucial for assessing material and energy cycling, nutrient limitation, and multi-element balance within biological systems. The relative ratios of C, N, and P determine plant nutrient-use efficiency and directly regulate ecosystem productivity and stability [3,4,5].

Forest floor material acts as a crucial interface linking plants and soil in forest ecosystems and serves as a reservoir of organic carbon and nutrients. Its decomposition dominates nutrient return to the soil, representing a primary pathway for sustaining forest nutrient cycling and soil fertility [6,7]. Additionally, as metabolic byproducts of plant tissues, forest floor material production and nutrient return rates directly regulate primary forest productivity. As a primary association in soil–plant nutrient cycling, forest floor material is essential for maintaining the health and stability of forest ecosystems [7,8]. Forest floor material stoichiometric traits reflect plant nutrient-use strategies and largely modulate microbial decomposition processes and nutrient release rates [4,9]. Therefore, understanding the distribution patterns of C, N, and P in forest floor material is crucial for elucidating the mechanisms underlying nutrient cycling in forest ecosystems. In forest ecosystems, the term “litter” is sometimes used ambiguously to refer either to freshly fallen plant material or to the organic layer accumulated on the surface of mineral soil [10]. To avoid such ambiguity, the term “forest floor material” is specifically employed in this study to denote the mixed organic materials collected from the surface organic layer, including recognizable and partially decomposed leaf litter, branch fragments, and other plant residues, which does not include freshly fallen litterfall. This material serves as a critical transitional pool in the nutrient cycling between plant organs and mineral soil. Therefore, “forest floor material” is adopted throughout the manuscript instead of “litter” to ensure terminological clarity.

Different tree species exhibit significant variations in nutrient uptake, allocation, and reabsorption strategies, resulting in differences in initial forest floor material quality [11,12]. Coniferous species tend to adopt a conservative nutrient strategy, characterized by forest floor material with high C/N and C/P ratios and slow decomposition rates. In contrast, broad-leaved species adopt a nutrient-enrichment strategy, with forest floor material having low C/N and C/P ratios that facilitates rapid nutrient release [13,14,15].Before senescence and abscission, nutrients such as N and P can be resorbed from leaves and transferred to perennial tissues [16]. Therefore, differences between living plant organs and forest floor material reflect not only decomposition processes but also nutrient resorption during senescence. Before forest floor material, plants regulate residual N and P levels in the forest floor material through nutrient reabsorption, thereby affecting forest floor material decomposition and nutrient cycling [17].

Allometric growth theory provides a crucial framework for understanding plant nutrient allocation and scaling relationships [18,19]. Specifically, the N–P scaling relationship is the primary indicator for assessing nutrient limitations in ecosystems. A significantly sub-unity N–P allometric slope typically indicates a stronger P limitation in the system [20]. In contrast, a slope not significantly different from 1 indicates isometric growth of N and P, reflecting a balanced nutrient supply or potential co-limitation. N–P scaling relationships can vary with organ type, plant functional groups, and environmental conditions [21]. In subtropical forests, the N–P allometric slopes of different organs (leaves, branches, and roots) did not significantly deviate from 1, indicating consistent isometric growth across organs. However, the deciduous and evergreen functional groups of subtropical shrubs exhibit distinct responses of N–P indices to light and soil nutrients [22]. Additionally, global change factors (precipitation and nitrogen deposition) may alter N–P scaling relationships [23]. Despite these advances, a systematic understanding of how N–P scaling relationships shift during the transformation of plant organs to forest floor material remains lacking—specifically in typical alpine ecosystems.

The southern slope of the Qilian Mountains is a typical alpine semi-arid ecological zone. Forest ecosystems play a crucial role in regional water conservation and ecological security [24,25]. Although previous studies have assessed soil nutrient distribution and forest floor material decomposition in this region [24,26,27,28,29,30], systematic analysis of C, N, and P stoichiometry and their allometric relationships in plant organs and forest floor material across dominant tree species remains limited—specifically regarding nutrient limitation mechanisms [31].

This study examined six representative tree species on the southern slope of the Qilian Mountains to analyze C, N, and P stoichiometry in plant organs, forest floor material, and their N–P scaling relationships. It addresses the following questions:

(1) Are there significant differences in nutrient allocation among plant organs and forest floor material across different tree species?
(2) Does the N–P scaling relationship shift during the transformation of plant organs to forest floor material?
(3) Can the N–P scaling relationship indicate the nutrient limitation pattern in this region?

## 2. Materials and Methods

### 2.1. Overview of the Study Area

The study area is located on the southern slope of the Qilian Mountains in northeastern Qinghai Province, China (102°06′–102°43′ E, 36°42′–37°06′ N), at the northeastern margin of the Qinghai-Tibet Plateau, where it forms a transitional zone between the Loess and Qinghai-Tibet Plateaus. The elevation ranges from 2118 to 4322 m, with a mean elevation of approximately 2710 m. This region has a typical alpine semi-arid climate, with a mean annual temperature of 5.8 °C and mean annual precipitation of approximately 477.4 mm [32]. The dominant soil types are gray-cinnamon soil and chestnut soil according to the Chinese soil classification system, which approximately correspond to Cambisols and Kastanozems in the World Reference Base for Soil Resources (WRB) classification, respectively [33], with higher humus content at elevations between 2500 and 3500 m [34]. Driven by the elevation gradient, vegetation types exhibit distinct vertical zonation, including coniferous, broad-leaved, and coniferous-broadleaved mixed forests [35]. This area is a significant water recharge zone for the Datong River, playing a crucial role in regional ecological security [36].

### 2.2. Sample Collection and Processing

Field surveys were conducted in August 2023. Six representative forest stands were selected based on vegetation type, including *Betula utilis*, *Sabina chinensis*, *Picea crassifolia*, *Picea asperata*, coniferous-broadleaved mixed, and broadleaved mixed forests. For each stand type, two independent replicate plots were established. Within each plot, three sampling points were randomly allocated, resulting in six sampling points.

At each sampling point, plant organ and surface forest floor material samples were collected separately. For plant organ sampling, living plant organs were sampled from healthy trees. Fully expanded, sun-exposed leaves from the upper-middle canopy and current-year branches were collected as representative living plant organ samples. To ensure representativeness, multiple individuals were sampled at each point, and the collected materials were pooled. For forest floor material sampling, the mixed organic layer on the surface of the mineral soil was collected. This material included leaf litter, branch fragments, and other partially decomposed or indistinguishable organic components. Thus, the sampled litter in this study refers to forest floor material rather than freshly fallen litterfall. All samples were transported to the laboratory, air-dried naturally, ground, sieved through a 100-mesh screen, sealed, and stored for subsequent analyses. Across the six stand types, six samples each of leaves, branches, and forest floor material were collected per stand, resulting in 36 samples for each category.

### 2.3. Determination of Nutrient Elements in Forest Floor Material

The C, N, P, and medium elements in plant organs (leaves and branches) and mixed forest floor material samples were determined using a unified method. The organic C content was determined using the potassium dichromate external heating method. Total N and P contents were determined using a flow analysis instrument and the molybdenum antimony colorimetric method, respectively, following sulfuric acid–hydrogen peroxide digestion. The total potassium (K) content was determined using an atomic absorption spectrophotometer after digestion. Water-soluble calcium (Ca) and magnesium (Mg) contents were extracted using a water extraction method and subsequently determined using an atomic absorption spectrophotometer [37,38,39,40].

### 2.4. Data Processing and Statistical Analysis

All statistical analyses were performed using SPSS 26.0 (IBM Corp., Armonk, NY, USA) and R (4.5.2 R Core Team, 2025). One-way analysis of variance (ANOVA) was used to assess differences in nutrient indices among tree species, followed by Tukey’s honestly significant difference post hoc test for multiple comparisons. Before ANOVA, the Shapiro–Wilk test was used to assess the data normality. Because the data violated the normality assumption, Spearman’s rank correlation analysis was conducted to assess the associations between nutrient element contents and stoichiometric ratios. Allometric relationships between N and P were analyzed using standardized major axis (SMA) regression analysis. Regression slopes were calculated using log-transformed data, and the significance of the deviations from a slope of 1 was tested. Principal component analysis (PCA) was applied to comprehensively characterize the differences in nutrient traits across tree species, with visualizations generated using Origin 2024 (OriginLab Corp., Northampton, MA, USA).

## 3. Results

### 3.1. Characteristics of C, N, and P Contents and Stoichiometric Ratios in Plant Organs and Forest Floor Material

There were significant differences in C, N, and P contents and their stoichiometric ratios among different tree species and components (*p* < 0.05, Table 1). The organic C content varied minimally across tree species, whereas the N and P contents exhibited significant interspecific differences. Compared with coniferous species, broad-leaved species and mixed forests had higher N and P contents in leaves, branches, and forest floor material.

Stoichiometric ratios highlight the differences in nutrient utilization strategies among tree species. Coniferous species consistently exhibited higher C/N and C/P ratios (120.55–131.99 and 1590.75–1752.30, respectively), whereas broad-leaved species and mixed forests exhibited lower C/N and C/P ratios (33.97–43.67 and 501.04–793.25, respectively). The C/N and C/P ratios were negatively associated with N and P contents, indicating that higher N and P contents were associated with lower ratios. Additionally, the N/P ratio exhibited relatively small variation across components, with broad-leaved species exhibiting higher N/P ratios than that of coniferous species. During the transformation from plant organs to forest floor material, both N and P contents were significantly reduced (*p* < 0.05), whereas the C/N and C/P ratios increased significantly.

### 3.2. Characteristics of K, Ca, and Mg Contents in Plant Organs and Forest Floor Material

There were significant differences in the K, Ca, and Mg contents among different tree species and components (*p* < 0.05, Table 2), and their variations were consistent with those of N and P. Overall, the K, Ca, and Mg contents in broad-leaved species and mixed forests were higher than those in coniferous species.

During the transformation from plant organs to forest floor material, the K, Ca, and Mg contents exhibited a reducing trend. Considering K as an example, the leaf K content of broad-leaved species and mixed forests ranged from 2.64 to 6.00 g·kg^−1^, branch K content from 15.24 to 20.14 g·kg^−1^, and forest floor material K content reduced from 2.46 to 6.46 g·kg^−1^. For coniferous species, leaf K content ranged from 0.47 to 3.89 g·kg^−1^, branch K content from 7.03 to 12.63 g·kg^−1^, and forest floor material K content reduced from 1.70 to 2.51 g·kg^−1^. Similar reductions were observed for Ca and Mg content.

### 3.3. Associations Between Nutrient Elements and Stoichiometric Ratios in Plant Organs and Mixed Forest Floor Material

Correlation analysis (Table 3) revealed a significant positive association (*p* < 0.01) between N and P across all organs. The strongest association occurred in forest floor material, followed by leaves and branches, indicating a consistent co-variation of N and P in plant organs. The relationships between organic C and N/P ratio varied by organ. In leaves, C was significantly negatively associated with both N and P (*p* < 0.01). In branches, C was significantly negatively associated with P (*p* < 0.01) but exhibited no significant association with N. In forest floor material, C was not significantly associated with either N or P (*p* > 0.05). This indicates that the trade-off between structural C and nutrients is most significant in the leaves. For stoichiometric ratios, C/N and C/P were significantly and positively associated (*p* < 0.01) across all organs. However, the association between C/P and N/P was not significant in leaves and forest floor material (*p* > 0.05), with a significant negative association observed only in branches (*p* < 0.05). Additionally, N was significantly negatively associated with C/N, and P was significantly negatively associated with C/P across all organs (*p* < 0.01).

### 3.4. Allometric Scaling Relationships Between Nutrient Elements in Plant Organs and Mixed Forest Floor Material

**Figure 1:**
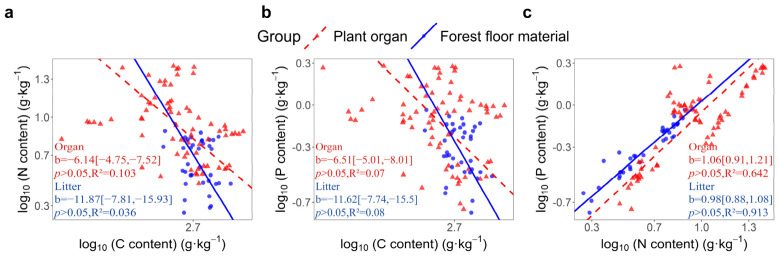
Allometric relationships between (**a**) carbon (C) and nitrogen (N), (**b**) carbon (C) and phosphorus (P), and (**c**) nitrogen (N) and phosphorus (P) concentrations in plant organs (red) and forest floor material (blue). All axes are log_10_-scaled. Lines represent standardized major axis (SMA) regressions. Corresponding statistical parameters are presented in the figure.

SMA regression analysis revealed significant allometric scaling relationships between nutrient elements in plant organs and forest floor material (Figure 1). For the C–N and C–P relationships, the C content remained relatively stable, whereas the N and P contents were significantly reduced during the transformation of plant organs to forest floor material—thereby increasing the C/N and C/P ratios.

In contrast, the N–P relationship exhibited distinct scaling characteristics. In plant organs, N and P contents exhibited a significant positive association, with an allometric scaling slope of 1.06 (95% confidence interval [CI]: 0.91–1.21)—this slope did not differ significantly from 1 (*p* > 0.05), and the coefficient of determination (R^2^) was 0.64. Additionally, for forest floor material, the N–P relationship was highly significantly positively associated with a more concentrated data distribution. Its allometric scaling slope was 0.98 (95% CI: 0.88–1.08)—that did not differ significantly from 1 (*p* > 0.05), and R^2^ = 0.91. The N–P allometric scaling slope in the forest floor material was comparable to that in plant organs, with highly overlapping CIs. During the transformation from plant organs to forest floor material, the allometric scaling slopes of N and P did not deviate significantly from 1.

### 3.5. Comprehensive Ordination of Nutrient Traits of Six Tree Species Based on PCA

**Figure 2:**
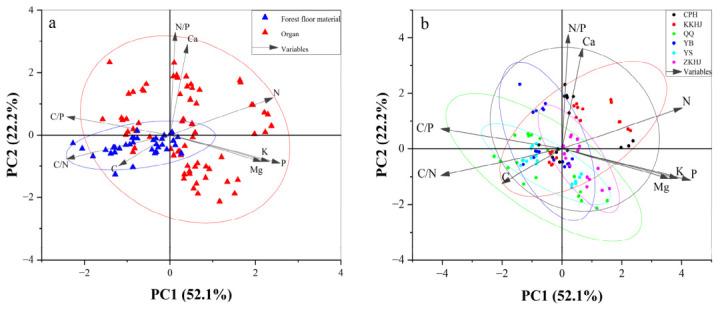
Principal component analysis of nutrient concentrations and stoichiometric ratios across different organs, forest floor material, and tree species. (**a**) Ordination of samples grouped by organ type and forest floor material. (**b**) Ordination of samples grouped by tree species. The abbreviations in the legend of Figure (**b**) denote the Pinyin initials of the following species and forest types: CPH, *Betula utilis* (Chinese common name: Caopihua); YB, *Sabina chinensis* (Chinese common name: Yuanbai); QQ, *Picea crassifolia* (Chinese common name: Qingqian); YS, *Picea asperata* (Chinese common name: Yunshan); ZKHJ, coniferous-broadleaved mixed forest (Chinese common name: Zhenkuohunjiaolin); KKHJ, broadleaved mixed forest (Chinese common name: Kuoyehunjiaolin). PC1 and PC2 explained 52.1 and 22.2% of the total variance, respectively. Points represent individual samples, while ellipses denote the 95% confidence intervals for each group, with the color of each ellipse corresponding to that of the respective group. Arrows represent variable loadings. All variables were standardized before analysis.

PCA revealed that the first two principal components cumulatively explained 74.30% of the total variance, with PC1 and PC2 accounting for 52.10 and 22.20% of the variance, respectively (Figure 2). At the organ level, leaves and forest floor material were distributed in the positive axis region of PC1, exhibiting positive associations with K, Ca, Mg, and P contents, but negative associations with C/N and C/P ratios. In contrast, woody organs were distributed in the negative axis, resulting in a systematic separation along PC1 (Figure 2a). At the tree species level, broad-leaved species and mixed forests were positioned on the positive PC1 axis, characterized by higher mineral nutrient contents and lower C/N and C/P ratios—indicating a nutrient enrichment strategy. Coniferous species were positioned on the negative PC1 axis, with higher C/N and C/P ratios—indicating a nutrient conservation strategy (Figure 2b). PC2 primarily captured variations in N/P ratios, and the distribution of tree species along this axis indicated persistent interspecific differences in N–P balance.

## 4. Discussion

### 4.1. Differences in the Chemical Stoichiometry of C, N, and P in Plant Organs and Forest Floor Material Among Different Tree Species and Their Mechanisms

This study demonstrated significant differences in C, N, and P contents and their stoichiometric ratios across plant organs and forest floor material among different tree species. These differences are closely associated with functional types of tree species. Coniferous species exhibited higher C/N and C/P ratios, whereas broad-leaved species and mixed forests exhibited lower C/N and C/P ratios—indicating a typical divergence between conservative and nutrient-enrichment nutrient utilization strategies. This aligns with previous research [13,14,41], indicating that tree species of different life forms adapt to environmental resource conditions through differentiated nutrient allocation and utilization strategies.

During the transformation from plant organs to forest floor material, the N and P contents are reduced significantly, whereas the C/N and C/P ratios are increased significantly—indicating a notable nutrient resorption before plant senescence [42,43]. The decrease in N and P concentrations from living plant organs to forest floor material may be explained by two processes. First, before leaf abscission, trees can resorb and retranslocate N and P from senescing leaves to perennial tissues such as branches, stems, and roots [44]. Second, after litter enters the forest floor, microbial decomposition and leaching may further alter nutrient concentrations [45]. Therefore, the observed differences between living organs and forest floor material should not be interpreted solely as decomposition loss, but rather as the combined result of nutrient resorption before abscission and post-depositional decomposition processes. This process may partly explain the lower N and P concentrations and higher C/N and C/P ratios observed in forest floor material. Compared with N, P was reduced more strongly during this process, indicating a higher P resorption efficiency, likely associated with the relative scarcity of P in alpine environments. In nutrient-limited ecosystems, plants sustain growth by enhancing nutrient resorption efficiency [3], and the findings of this study further support this proposition.

Interspecific differences in nutrient utilization strategies among tree species may modulate forest floor material decomposition and nutrient cycling by altering forest floor material quality. Broad-leaved species and mixed forests, characterized by higher N and P contents, produce forest floor material more readily decomposed by microorganisms, thereby facilitating rapid nutrient release. In contrast, coniferous forest floor material, characterized by higher C/N and C/P ratios and a greater proportion of recalcitrant components, decomposes more slowly. These differences partially account for the functional divergence of different forest stand types in nutrient cycling.

### 4.2. Analysis of Nutrient Limitation and Utilization Strategies Based on Allometric Growth Relationships

Compared with plant organs, forest floor material exhibits similar N–P allometric slopes with highly overlapping confidence intervals, indicating that nutrient resorption during the transformation from plant organs to forest floor material does not significantly alter N–P stoichiometric coupling. This finding differs from that of Kerkhoff et al. [11] who analyzed global seed plants and reported leaf N–P allometric slopes generally <1, attributing this pattern to plant growth rate and nutrient limitation. Yuan et al. [17] observed latitudinal variation in leaf N–P allometric slopes on a global scale, although most remained below 1. However, Chen et al. [21] analyzed 224 woody plant species across multiple organs (leaves, branches, and fine roots) in subtropical forests and observed that the N–P allometric slopes of all organs did not significantly deviate from 1, with a common slope of 1.08—highly consistent with our results. Wang et al. [46] reported a shared isometric growth relationship (allometric exponent = 1) between N and P in the needles, stems, and roots of seedlings from nine spruce taxa. These studies indicate that isometric growth is relatively prevalent across climate zones and plant functional types, and a slope < 1 may not represent the sole pattern.

The isometric growth observed in this study can be attributed to the following mechanisms. First, the alpine ecosystem on the southern slope of the Qilian Mountains is characterized by a short growing season and low temperatures, resulting in relatively slow plant growth. According to the growth rate hypothesis, rapid growth requires large quantities of P-rich rRNA, resulting in N–P slopes < 1. In contrast, slow growth reduces P demand, potentially driving the N–P slopes toward 1. In *Arabidopsis* fertilization experiments, N and P supply levels significantly regulate N–P allometric relationships—when the P supply is not absolutely limiting, these relationships may exhibit isometric or near-isometric patterns [47]. Second, intense nutrient competition may occur between plants and soil microorganisms. Microbial P immobilization can counteract plant P resorption effects, thereby maintaining stable N:P ratios in the forest floor material. Third, the forest floor material samples in this study were mixed (leaves and branches). Variations in resorption efficiency across different organs may have been averaged during sample mixing, masking organ-specific P depletion trends.

Soil P availability on the southern slope of the Qilian Mountains may not be extremely low under the regional environmental conditions. Bai et al. [24] assessed the soil ecological stoichiometry of typical vegetation in the Qilian Mountains, with total soil P content ranging from 0.41 to 0.66 g·kg^−1^ and available P content between 4.35 and 13.57 mg·kg^−1^. This indicates that soil P availability in this region is moderate and may not have reached the threshold triggering a strong P limitation response. Additionally, Qin et al. [48] studied the leaf stoichiometry of grassland plants in the Dayekou Basin of the Qilian Mountains and observed that nutrient limitation types varied across slope aspects. Plants on the southern slope were P-limited, whereas those on the northern slope were N-limited. This indicates that within the same region, local environmental factors, such as slope aspect can significantly shape nutrient limitation patterns. Therefore, in the forest ecosystem, plant growth may be co-limited by N and P or primarily regulated by other environmental factors (low temperature and growing season length), rather than being solely P-limited.

Additionally, C/N exhibited a highly significant positive association with C/P, whereas N exhibited a highly significant negative association with C/N and P with C/P (Table 3). This indicates that forest floor material stoichiometric ratios are primarily governed by nutrient element content, confirming the central role of N and P in forest floor material decomposition and nutrient cycling [9,31].

Overall, this study revealed interspecific differences in nutrient utilization based on allometric growth relationships and demonstrated that N–P exhibited isometric growth in this forest ecosystem, with no evidence of significant P limitation. These findings provide a novel scientific basis for nutrient management in alpine forests, highlighting the need to prioritize the coordinated supply of N and P rather than focusing solely on P supplementation.

### 4.3. Effect of Chemical Stoichiometry of Forest Floor Material on Nutrient Cycling in Ecosystems and Its Significance

In this study, the C/N and C/P ratios of coniferous forest floor material (125.87–235.71 and 1181.45–2006.45, respectively) were significantly higher than those of broadleaf and mixed forest floor material (C/N: 78.81–87.12 and C/P: 729.11–798.13). This variation primarily arises from the distinct nutrient-use strategies among plant functional types. Conifers exhibit a conservative strategy, characterized by high N and P resorption efficiency before forest floor material to retain nutrients in vivo, resulting in low N and P concentrations in the forest floor material. In contrast, broadleaf species and mixed forests adopt a nutrient-enrichment strategy with higher forest floor material N and P concentrations and lower C/N and C/P ratios (Table 1). Additionally, coniferous forest floor material contains higher levels of recalcitrant components (lignin and tannins) that further increase its C/N and C/P ratios.

These distinct stoichiometric traits of forest floor material have divergent implications for decomposition and nutrient cycling. First, high C/N and C/P ratios are typically associated with slower forest floor material decomposition rates because microorganisms may face N or P limitation during the early decomposition stage [49]. In this study, the C/P ratio of coniferous forest floor material reached 1590–2006, far exceeding the global average for forest floor material (approximately 300–600), indicating that its decomposition can be chronically constrained by relative P limitation.

However, this study revealed that the N–P allometric relationships in both plant organs and forest floor material had slopes not significantly different from 1, indicating isometric growth—where the N:P stoichiometric ratio remained constant, with no absolute P deficiency detected. This result indicates that the relative P limitation reflected by high C/P ratios is C-dependent, whereas the stoichiometric coupling between N and P remains intact. When decomposing high C/P forest floor material, microorganisms may require additional P from the environment; however, this demand did not reach a threshold sufficient to alter the N–P allometric relationship.

In contrast, the C/N and C/P ratios serve as indicators of nutrient return potential. The lower C/N and C/P ratios of broadleaf and mixed-forest floor material indicate higher decomposability by microorganisms and faster nutrient release rates, facilitating the rapid replenishment of soil available nutrients. In contrast, the slow decomposition of coniferous pure-forest floor material results in the long-term immobilization of nutrients in the forest floor material layer, potentially reducing soil nutrient availability and establishing a positive feedback loop of nutrient limitation.

From the perspective of ecological restoration implications, PCA in this study (Figure 2) clearly classified tree species into two functional groups—nutrient-enriching and conservative—consistent with their forest floor material stoichiometric traits. In the alpine ecosystem on the southern slope of the Qilian Mountains, large-scale afforestation of coniferous monocultures may result in low-quality forest floor material, slow decomposition, and retarded nutrient cycling. In contrast, establishing coniferous–broadleaf mixed forests—by introducing broadleaf species to enhance the overall quality of forest floor material—can alleviate nutrient limitation during decomposition, enhance microbial activity, and facilitate the coordinated release of N and P. Therefore, ecological restoration should prioritize the development of coniferous–broadleaf mixed forests, focusing on coordinated N and P supply rather than exclusive P supplementation.

Limitations of the present study are as follows: Senescent leaves and freshly fallen forest floor material were not collected separately, which precluded the quantitative differentiation between nutrient resorption prior to plant organ abscission and the decomposition and leaching processes subsequent to litter deposition. Additionally, the mineral carbon (C), nitrogen (N), and phosphorus (P) in soil were not analyzed synchronously, constraining our ability to directly evaluate the impact of soil nutrient supply on the stoichiometric relationships of plant organs and forest floor material. Furthermore, soil nutrient availability was not assessed concurrently, which restricts our capacity to directly link the stoichiometric patterns in plants and forest litter to soil nutrient supply—a factor that may influence both plant nutrient uptake and the nutrient properties of litter.

For future research, it is recommended to incorporate samples of live leaves, senescent leaves, fresh litter, forest floor material, and soil mineral fractions to more explicitly elucidate nutrient translocation pathways in alpine forest ecosystems. Additionally, integrating data on soil mineral nutrient measurements will enable more precise discrimination between species effects and soil nutrient effects.

## 5. Conclusions

In the southern slope region of the Qilian Mountains, significant variations in the stoichiometric characteristics of carbon (C), nitrogen (N), and phosphorus (P) are observed across different tree species, plant organs, and forest floor material. Broadleaf plants generally display higher nutrient concentrations, while coniferous plants tend to have higher C/N and C/P ratios. In comparison to living plant organs, forest floor material exhibits lower N and P concentrations but higher C/N and C/P ratios; this phenomenon may reflect the combined effects of nutrient resorption occurring prior to plant organ abscission and decomposition or leaching processes taking place after litter deposition; however, the N–P allometric slope remained unaltered (1.06 and 0.98 for plant organs and forest floor material, respectively), with neither differing significantly from 1. The positive N–P relationship suggests relatively coordinated N and P variation and indicates that P availability may not be the primary limiting factor in the studied forest ecosystems.

From plant organs to mixed forest floor material, N and P concentrations were significantly reduced, whereas C/N and C/P ratios were significantly increased, indicating that nutrient resorption is the primary mechanism regulating forest floor material stoichiometric traits. N and P exhibited highly significant positive associations in both plant organs and forest floor material, with N–P allometric slopes not deviating significantly from 1 (isometric growth), indicating that N and P maintain a relatively constant ratio throughout plant growth and forest floor material decomposition. Notably, coniferous forest floor material exhibited extremely high C/P ratios (1590–2006); however, its N–P slope did not fall below 1. A high C/P ratio indicates an absolute imbalance between C and P, whereas the N–P allometric slope characterizes the coupling of relative change rates between N and P—these two metrics correspond to distinct dimensions of nutrient limitation. PCA further categorized tree species into nutrient-enriching and conservative functional groups, strongly supporting the significant divergence in nutrient-use strategies among different functional tree types. These findings reveal the regional specificity of N–P synergistic coupling in the forest ecosystem on the southern slope of the Qilian Mountains, providing a scientific basis for alpine forest ecological restoration. Priority should be given to establishing coniferous–broadleaf mixed forests, with an emphasis on the synergistic supply of N and P rather than exclusive P supplementation, thereby enhancing nutrient cycling efficiency and ecosystem stability.

## Figures and Tables

**Table 1:** Carbon (C), nitrogen (N), and phosphorous (P) concentrations and stoichiometric ratios of plant organs and forest floor material across different species.

Component	Species	C (g·kg^−1^)	N (g·kg^−1^)	P (g·kg^−1^)	C/N	C/P	N/P
Leaf	*Betula utilis*	473.32 ± 5.80 bc	11.58 ± 0.30 b	0.60 ± 0.03 b	41.05 ± 1.39 b	793.25 ± 43.01 b	19.29 ± 0.66 a
Leaf	*Juniperus przewalskii*	483.06 ± 11.08 bc	4.11 ± 0.30 d	0.29 ± 0.03 c	120.55 ± 8.58 a	1752.3 ± 235.19 a	14.4 ± 1.17 bc
Leaf	*Picea crassifolia*	500.03 ± 4.12 ab	4.10 ± 0.36 d	0.32 ± 0.04 c	126.99 ± 11.47 a	1699.34 ± 223.56 a	13.17 ± 0.70 c
Leaf	Mixed forest	406.19 ± 10.81 d	9.30 ± 0.15 c	0.82 ± 0.03 a	43.67 ± 0.64 b	501.04 ± 27.31 b	11.46 ± 0.53 c
Leaf	*Picea asperata*	532.71 ± 5.72 a	4.04 ± 0.04 d	0.34 ± 0.02 c	131.99 ± 2.33 a	1590.75 ± 76.55 a	12.06 ± 0.59 c
Leaf	Broadleaf mixed forest	459.41± 5.77 c	13.57 ± 0.33 a	0.82 ± 0.02 a	33.97 ± 1.09 b	563.79 ± 19.56 b	16.6 ± 0.29 ab
Branch	*Betula utilis*	476.48 ± 12.25 ab	24.17 ± 0.44 a	1.82 ± 0.03 a	19.76 ± 0.73 c	262.66 ± 9.68 d	13.30 ± 0.26 a
Branch	*Juniperus przewalskii*	540.03 ± 8.70 a	7.78 ± 0.21 d	0.89 ± 0.02 d	69.72 ± 2.52 a	607.49 ± 22.99 a	8.72 ± 0.14 b
Branch	*Picea crassifolia*	472.76 ± 22.71 b	6.60 ± 0.17 d	1.34 ± 0.18 bc	71.90 ± 4.22 a	388.59 ± 55.78 cd	5.31 ± 0.61 c
Branch	Mixed forest	476.15 ± 17.50 ab	9.89 ± 0.46 c	1.21 ± 0.11 bcd	48.73 ± 3.19 b	410.12 ± 38.82 bc	8.35 ± 0.36 b
Branch	*Picea asperata*	514.25 ± 1.53 ab	7.26 ± 0.19 d	0.96 ± 0.02 cd	70.91 ± 2.29 a	534.75 ± 20.47 ab	7.55 ± 0.11 b
Branch	Broadleaf mixed forest	465.52 ± 4.62 b	19.62 ± 0.65 b	1.38 ± 0.07 b	23.86 ± 0.83 c	341.44 ± 18.76 cd	14.27 ± 0.31 a
Forest floor material	*Betula utilis*	496.08 ± 8.34 a	6.62 ± 0.59 a	0.72 ± 0.07 a	79.39 ± 10.14 d	729.11 ± 87.60 c	9.21 ± 0.18 a
Forest floor material	*Juniperus przewalskii*	507.23 ± 6.75 a	4.29 ± 0.57 bc	0.46 ± 0.06 bc	125.87 ± 12.11 bc	1181.45 ± 114.66 bc	9.41 ± 0.30 a
Forest floor material	*Picea crassifolia*	505.71 ± 7.14 a	2.21 ± 0.18 d	0.29 ± 0.05 c	235.71 ± 17.48 a	2006.45 ± 306.92 a	8.32 ± 0.83 a
Forest floor material	Mixed forest	508.16 ± 4.02 a	6.46 ± 0.11 a	0.68 ± 0.04 a	78.81 ± 1.39 d	763.36 ± 43.20 c	9.68 ± 0.49 a
Forest floor material	*Picea asperata*	523.93 ± 7.32 a	3.18 ± 0.10 cd	0.33 ± 0.01 c	165.90 ± 6.79 b	1598.69 ± 62.93 ab	9.65 ± 0.17 a
Forest floor material	Broadleaf mixed forest	500.15 ± 9.06 a	5.75 ± 0.05 ab	0.63 ± 0.02 ab	87.12 ± 1.91 cd	798.13 ± 32.53 c	9.17 ± 0.37 a

Note: Data in the table are expressed as the mean ± standard deviation (n = 3). Within the same component, different lowercase letters in the same column indicate significant differences (*p* < 0.05).

**Table 2:** Concentrations of K, Ca, and Mg in plant organs and forest floor material across different species.

Component	Species	K (g·kg^−1^)	Ca (g·kg^−1^)	Mg (g·kg^−1^)
Leaf	*Betula utilis*	2.20 ± 0.21 c	1.07 ± 0.05 b	0.33 ± 0.01 c
Leaf	*Juniperus przewalskii*	2.84 ± 0.13 bc	1.49 ± 0.04 a	0.24 ± 0.02 d
Leaf	*Picea crassifolia*	3.89 ± 0.46 b	0.71 ± 0.05 c	0.13 ± 0.01 e
Leaf	Mixed forest	6.00 ± 0.71 a	0.61 ± 0.02 c	0.27 ± 0.01 d
Leaf	*Picea asperata*	0.47 ± 0.12 d	0.66 ± 0.04 c	0.49 ± 0.01 a
Leaf	Broadleaf mixed forest	2.64 ± 0.09 bc	0.97 ± 0.06 b	0.39 ± 0.01 b
Branch	*Betula utilis*	20.14 ± 0.86 a	0.87 ± 0.04 b	0.64 ± 0.01 c
Branch	*Juniperus przewalskii*	7.07 ± 0.24 d	0.82 ± 0.03 b	0.45 ± 0.02 d
Branch	*Picea crassifolia*	12.63 ± 1.45 c	0.39 ± 0.05 c	0.59 ± 0.03 c
Branch	Mixed forest	15.24 ± 0.89 bc	0.42 ± 0.02 c	0.70 ± 0.00 b
Branch	*Picea asperata*	7.03 ± 0.72 d	0.53 ± 0.02 c	0.72 ± 0.01 ab
Branch	Broadleaf mixed forest	17.64 ± 1.68 ab	1.44 ± 0.10 a	0.77 ± 0.00 a
Forest floor material	*Betula utilis*	2.51 ± 0.28 b	0.77 ± 0.06 b	0.24 ± 0.01 bcd
Forest floor material	*Juniperus przewalskii*	2.10 ± 0.16 b	0.66 ± 0.05 bc	0.17 ± 0.02 d
Forest floor material	*Picea crassifolia*	1.70 ± 0.41 b	0.52 ± 0.03 c	0.18 ± 0.02 cd
Forest floor material	Mixed forest	6.46 ± 0.46 a	0.93 ± 0.05 a	0.53 ± 0.02 a
Forest floor material	*Picea asperata*	1.98 ± 0.28 b	0.62 ± 0.02 bc	0.25 ± 0.02 bc
Forest floor material	Broadleaf mixed forest	2.46 ± 0.21 b	0.63 ± 0.03 bc	0.28 ± 0.02 b

Note: Data in the table are expressed as the mean ± standard deviation (n = 3). Within the same component, different lowercase letters in the same column indicate significant differences (*p* < 0.05).

**Table 3:** Correlation analysis of nutrient elements and stoichiometric ratios of plant organs and forest floor material.

Component	Indicators	C	N	P	C/N	C/P	N/P
Leaf	C	1					
	N	−0.544 **	1				
	P	−0.753 **	0.877 **	1			
	C/N	0.705 **	−0.944 **	−0.922 **	1		
	C/P	0.696 **	−0.832 **	−0.931 **	0.927 **	1	
	N/P	0.071	0.527 **	0.088	−0.373 *	−0.048	1
Branch	C	1					
	N	−0.317	1				
	P	−0.528 **	0.726 **	1			
	C/N	0.517 **	−0.942 **	−0.728 **	1		
	C/P	0.730 **	−0.691 **	−0.940 **	0.771 **	1	
	N/P	−0.134	0.882 **	0.346 *	−0.842 **	−0.352 *	1
Forest floor material	C	1					
	N	−0.199	1				
	P	−0.294	0.959 **	1			
	C/N	0.240	−0.934 **	−0.897 **	1		
	C/P	0.329	−0.865 **	−0.902 **	0.937 **	1	
	N/P	0.322	0.178	−0.089	−0.154	0.173	1

Note: ** denotes statistical significance at the 0.01 level (two-tailed), whereas * denotes statistical significance at the 0.05 level (two-tailed). The sample size for each component is N = 36.

## Data Availability

The original contributions presented in this study are included in the article. Further inquiries can be directed to the corresponding authors.
